# Antimicrobial mechanisms and secondary metabolite profiles of *Streptomyces hygroscopicus* subsp. *hygroscopicus* 5–4 against banana fusarium wilt disease using metabolomics

**DOI:** 10.3389/fmicb.2023.1159534

**Published:** 2023-06-09

**Authors:** Tianyan Yun, Tao Jing, Xiaoping Zang, Dengbo Zhou, Kai Li, Yankun Zhao, Wei Wang, Jianghui Xie

**Affiliations:** ^1^National Key Laboratory for Tropical Crop Breeding, Hainan Institute for Tropical Agricultural Resources, Chinese Academy of Tropical Agricultural Sciences (CATAS), Haikou, China; ^2^Haikou Experimental Station, Chinese Academy of Tropical Agricultural Sciences (CATAS), Haikou, China

**Keywords:** fusarium wilt of banana, *Streptomyces hygroscopicus* subsp. *hygroscopicus*, antifungal mechanism, metabolomics, hygromycin B

## Abstract

Fusarium wilt of bananas (FWB) is seriously affecting the sustainable development of the banana industry and is caused by the devastating soil-borne fungus *Fusarium oxysporum* f. sp. *cubense* tropical race 4 (Foc TR4). Biological control is a promising strategy for controlling Fusarium wilt in bananas. We previously identified *Streptomyces hygroscopicus* subsp. *hygroscopicus* 5–4 with strong antifungal activity against the FWB. The most possible antimicrobial mechanism of strain 5–4 was explored using the metabolomics approach, light microscopy imaging, and transmission electron microscopy (TEM). The membrane integrity and ultrastructure of Foc TR4 was damaged after extract treatment, which was supported by the degradation of mycelium, soluble protein content, extracellular reducing sugar content, NADH oxidase activity, malondialdehyde content, mitochondrial membrane potential, and mitochondrial respiratory chain complex enzyme activity. The extracts of strain 5–4 cultivated at different times were characterized by a liquid chromatography–mass spectrometer (LC-MS). 647 known metabolites were detected in the extracts of strains 5–4. Hygromycin B, gluten exorphin B4, torvoside G, (z)-8-tetradecenal, piperitoside, sarmentosin, pubescenol, and other compounds were the main differential metabolites on fermentation culture for 7 days. Compared with strain 5–4 extracts, hygromycin B inhibited the mycelial growth of Foc TR4, and the EC_50_ concentration was 7.4 μg/mL. These results showed that strain 5–4 could destroy the cell membrane of Foc TR4 to inhibit the mycelial growth, and hygromycin B may be the key antimicrobial active metabolite. *Streptomyces hygroscopicus* subsp. *hygroscopicus* 5–4 might be a promising candidate strain to control the FWB and provide a scientific basis for the practical application of hygromycin B as a biological control agent.

## Introduction

Banana is an important fruit in tropical and subtropical regions worldwide (Qi et al., 2021). Due to the fusarium wilt of bananas (FWB), the high morbidity, great depredation, and rapid spread led to a sharp reduction in the area under traditional banana cultivation (Dita et al., 2018; Yun et al., 2022). FWB, caused by the devastating soil-borne fungus *Fusarium oxysporum* f. sp. *cubense* tropical race 4 (Foc TR4), is seriously affecting the sustainable development of the banana industry (Prigigallo et al., 2022). Biocontrol was considered to be one of the most effective methods compared with different prevention strategies (Fu et al., 2017). This provides new opportunities for FWB control (Du et al., 2022). Biocontrol microbes such as *Pseudomonas* sp., *Bacillus* sp., and *Streptomyces* sp. have been reported to be used in controlling the FWB (Bubici et al., 2019). Although many biocontrol microbes had a good inhibitory effect on Foc *in vitro*, their control effect was often limited and unstable in the field due to the different factors of soil environmental conditions, such as pH, osmotic pressure, and salt concentration (Du et al., 2022). Therefore, it is still necessary to adapt to local conditions to develop more safe and effective biological agents.

Actinomycetes, especially *Streptomyces* with biocontrol potential, are deemed significant producers of bioactive metabolites. *Streptomyces* are believed to be unique among rhizosphere microorganisms in terms of disease management (Kaari et al., 2022). They also exist as potential endophytic microorganisms, and most colonize the tissues of plants without causing any negative effects (Kaari et al., 2022). Therefore, endophytic *Streptomyces* was considered one of the most promising strategies for the biocontrol of plant pathogens (Xu et al., 2020). In general, biocontrol microorganisms protect plants against phytopathogens through direct and indirect mechanisms. Direct mechanisms for microbial assistance against pathogens include antibiosis, lytic enzymes, hyperparasitism, predation, and competition for nutrients and space (Xu et al., 2019; Ebrahimi et al., 2022). For example, *Streptomyces corchorusii* AUH-1 could damage the cell membranes of pathogens by inhibiting ergosterol formation and increasing malondialdehyde levels (Yang et al., 2019). In Foc TR4 hyphae treated with endophytic *Streptomyces* sp. 5–10 extracts, the cell ultrastructure and membrane integrity were damaged (Yun et al., 2021). In our previous study, *Streptomyces hygroscopicus* 5–4 can secrete chitinase and β-1,3-glucanase, thus damaging the cell wall of Foc TR4 (Yun et al., 2022). In addition to the cell wall, the cell membrane is the mechanical and osmotic barrier between the cell and the environment and is a common action site of fungicides against pathogenic fungi (Kim and Lee, 2019; Xu et al., 2019; Zhang et al., 2021). To reveal the possible action mode of strain 5–4 in inhibiting Foc TR4, this study focused on the effect of strain 5–4 extracts on Foc TR4 cell membranes and the antifungal metabolic profiles of strain 5–4. The main aims of this study are as follows: (i) to reveal the possible underlying mechanism of action by studying the integrity of the cell membrane and microscopic morphology of mycelia and (ii) to determine the metabolic profiles of strain 5–4 in different fermentation culture periods by metabolomic analyses.

## Materials and methods

### Microorganisms and culture media

*Streptomyces hygroscopicus* subsp. *hygroscopicus* 5–4 (GDMCC 61679) was isolated from the roots of *Piper austrosinense* and maintained on the International *Streptomyces* Program 2 (ISP2) medium at 4°C. Banana wilt disease pathogenic fungi *Fusarium oxysporum* f. sp. *cubense* tropical race 4 (Foc TR4, ATCC 76255) were kept on a Petri dish containing potato dextrose agar (PDA) at 4°C.

### Antagonistic effects of strain 5–4 on mycelial development of Foc TR4 *in vitro*

Strain 5–4 was able to effectively inhibit the growth of Foc TR4 mycelium, as determined using a modified method of confrontation culture assay of detection (Getha and Vikineswary, 2002). Briefly, a sterilized cover slip (1 × 1 cm) was placed at the center of the PDA medium and inoculated with Foc TR4 and strain 5–4 at both ends of the coverslip, respectively. After being cultivated for 5 days at 28°C, the coverslips were observed using a microscope (model Axio Scope A1, Carl Zeiss AG, Germany).

### Strain cultivation and metabolite extraction

Pre-cultures: strain 5–4 was inoculated in ISP2 medium and cultivated for 3 days at 28°C. Strain cultivation: 3 mL of pre-culture was inoculated in 100 mL of soybean liquid culture medium (SLM) and cultivated for 7 days at 28°C at 200 rpm (Chen et al., 2018). The strain 5–4 metabolites were extracted with 98% absolute ethanol (filtrate: ethanol = 1:1 vol/vol) from the cultural liquid. After being evaporated in a rotary vacuum evaporator, the extracts were dissolved in distilled water and dried by vacuum freezing and drying technology (Yun et al., 2022). The extracts of strain 5–4 were stored at 4°C.

### Effect of extracts on Foc TR4 mycelial *in vitro*

A fungal disk of 0.5 mm diameter was inoculated on the PDA plate. The sterilized coverslips were placed near the fungal disk. When the fungal mycelia were overgrown with coverslips, the coverslips were removed and placed in sterile Petri dishes, then added 50 μL of strain 5–4 extracts to the coverslips at a concentration of 500 μg/mL (Yun et al., 2022). The mycelial morphology of Foc TR4 was investigated under a microscope after treatment at 28°C for 2 days.

### Effect of extracts on the ultrastructure of Foc TR4 cells

The Foc TR4 was inoculated on the PDA plate, which contained a final concentration of 500 μg/mL extracts. After being cultured at 28°C for 5 days and collected from the edge of the inhibition zone for the Foc TR4 mycelia, the same volume of sterile water was used as a negative control. Samples were fixed, dehydrated, and embedded into Epon 812 resin according to the methods of Cao et al. (2022). The Foc TR4 mycelia were sliced by an ultra-thin slicer (Leica, UC6 CM1950, Germany). The ultrastructure of the mycelial sample was observed using a transmission electron microscope (TEM, HT7700, Hitachi, Ibaraki, Japan).

### Effect of extracts on the cell membrane of Foc TR4

In addition to the cell wall, the integrity of the cell membrane is also another key factor for normal microbial life (Xu et al., 2019). Thus, the effect of extracts on the cell membrane integrity of Foc TR4 was detected.

#### Effects of extracts on the content of soluble protein (SP) in Foc TR4 mycelia

Briefly, an aliquot (1 mL) of Foc TR4 spore suspension (1 × 10^5^ per/mL) was added to a 30 mL PDB and cultured at 28°C for 5 days. The fungal mycelia were centrifuged at 4000 rpm for 15 min and washed three times with sterilized water (Wang et al., 2022). A total of 0.5 g of the samples was weighed and resuspended in 10 mL of sterilized water. The extracts were added to the resuspended solution to achieve final concentrations of EC_50_ (62.79 μg/mL) and EC_95_ (869.94 μg/mL), which were obtained in previous studies (Yun et al., 2022). Subsequently, the samples were incubated for 0, 2, 4, 8, 12, and 24 h at 28°C on a rotary shaker at 200 rpm and centrifuged at 4000 rpm for 10 min to obtain supernatants. Sterile water treatment was used as a negative control. The content of SP was determined using the Bradford (1976) method based on bovine serum albumin (BSA) as the standard. Each treatment was performed in triplicate.

#### Effects of extracts on the content of extracellular reducing sugar (ERS) in Foc TR4 mycelia

The mycelia of Foc TR4 were cultured as described above, incubated for 3, 6, 12, and 24 h at 28°C on a rotary shaker at 200 rpm, and centrifuged at 4000 rpm for 10 min to obtain supernatants. Then, we added 0.18 mL of DNS reagent for each 0.24 mL of the supernatant. The content of ERS was determined using the 3,5-dinitrosalicylic (DNS) colorimetric method according to Yuan et al. (2014). All experiments were performed in triplicate.

#### Effects of extracts on the content of NADH oxidase (NOX) activity in Foc TR4 mycelia

The samples were cultured using the abovementioned method. The mycelia were washed two times with PBS; 0.1 g of the sample was ground, and the powdered tissue was dissolved in 1 mL of extraction solution, and the mixture was centrifuged at 8000 rpm for 10 min to collect supernatants.

The NADH oxidase activity content was determined using the NADH oxidase activity detection kit (Beijing Solebao Technology Inc., Beijing, China) (Chen et al., 2018).

#### Effects of extracts on the content of malondialdehyde (MDA) in Foc TR4 mycelia

The membrane lipid peroxidation can be analyzed by determining the content of MDA. The mycelia of Foc TR4 were cultured as described above. According to the kit instructions (the MDA kit from Beijing Solebao Technology Inc., Beijing, China), the activity of MDA was determined (Wang et al., 2019).

### Effect of extracts on the mitochondrial membrane potential of Foc TR4

When Rhodamine-123 is applied to intact cells, it is predominantly localized in mitochondria and represents a reliable fluorescent probe for assessing the mitochondrial membrane potential (Anamika et al., 2021). The spore suspension of Foc TR4 (10^6^ per/mL) was equally mixed with strain 5–4 extracts and incubated at 28°C for 2 h (Jiang et al., 2021; Yun et al., 2022). The samples were washed two times with PBS and incubated with cationic lipophilic rhodamine-123 at 37°C for 30 min (Zhu et al., 2012). Sterile water was used as a negative control. Effect on the mitochondrial membrane potential of Foc TR4 with extracts observed using fluorescence microscopy (MMI Cell Cut Plus, Leica DM6000B, Wetzlar, Germany).

### Effect of extracts on mitochondrial respiratory chain complex enzyme activity of Foc TR4

Spore suspensions of Foc TR4 (10^6^ per/mL) were incubated in a PDB liquid medium at 28°C for 5 days. The mycelia of Foc TR4 were then collected by centrifugation at 4000 r/min for 15 min and washed three times with sterile water. An ice bath was used to grind an appropriate amount of mycelium, a mitochondrial buffer, and quartz sand. The resulting homogenate was further centrifuged at 2000 rpm for 10 min at 4°C to collect the supernatants (Li et al., 2022). The supernatants were centrifuged at 12,000 rpm for 15 min, and the resulting sediments were retained. A proper amount of buffer was added to the sediments to make them fully suspended, followed by another centrifugation at 12,000 rpm for 15 min to collect the sediments, which were identified as mitochondria.

The protein content of mitochondria was adjusted to 50 μg/mL (Li et al., 2022). We then added the extracts of strain 5–4 to the mitochondria, and the final concentrations were EC_50_ (62.79 μg/mL) and EC_95_ (869.94 μg/mL), respectively. The samples were incubated at 37°C for 30 min. A negative control consisting of sterile water was included for comparison purposes. Enzyme activity assays of NADH CoQ reductase (mitochondrial complexes I), succinate dehydrogenase (mitochondrial complexes II), cytochrome oxidase (mitochondrial complexes III), and F_1_F_0_-ATP synthase (mitochondrial complexes V) were performed using the Assay Kit (Solarbio Beijing, China) (Zhao et al., 2020). Each treatment group was repeated three times.

### Analysis of metabolites from strain 5–4 during different culture time points by HPLC-MS

To find the metabolites with anti-Foc RT4 activity during the fermentation of strain 5–4, we analyzed the changes in metabolites under different culture times by metabolomics.

#### Preparation of samples

Strain 5–4 was initially cultivated in 100 mL of SLM medium. After being cultivated for the 2nd, 4th, 6th, 7th, and 8th days on a rotary shaker for 180 rpm at 28°C, the metabolites were extracted with methyl alcohol (filtrate: methyl alcohol = 1:4 vol/vol) from the cultural liquid. The methyl alcohol extracts were concentrated in vacuo to obtain the dried material. The antimicrobial activity was evaluated against Foc TR4 using the filter paper diffusion method (Yun et al., 2022) to determine the fermentation culture time points for HPLC-MS detection.

#### HPLC-MS

The LC-MS analysis was performed using the UHPLC-Q exertive system by Thermo Fisher Scientific. The separation was carried out using an HSS T3 column (100 mm × 2.1 mm i.d., 1.8 μm), and the eluted samples were subsequently directed into mass spectrometry detection for analysis (Yang et al., 2022; Zhang et al., 2023). The mobile phase was (A) water with 0.1% of formic acid and (B) acetonitrile/isopropanol (1:1 vol/vol) with 0.1% of formic acid (Yang et al., 2022). The samples were determined using HPLC-MS spectrometry and injected with 0.4 mL/min of mobile phase; the injection volume was 2 μL. The column temperature was 40°C. The solvent gradient started at 5% A for 3 min, was converted to 95% B for 9 min, and was maintained at 95% B for 13 min. Sample-quality spectrum signals were collected by positive and negative ion scanning modes and an electrospray ionization source. The capillary voltages of positive mode, negative mode, sheath gas flow rate, aus gas flow rate, aus gas heater temp, and normalized collision energy were set to be 3.5, 2.8 kV, 40 psi, 10 psi, 400°C, and 20-40-60 V, respectively. Data were collected in a profile mode from 70 to 1050 m/z.

#### Data processing

The response intensity of the sample mass spectrum peaks was normalized using the method of sum normalization and obtained as the normalized data matrix (Kaleem et al., 2022). The variables with a relative standard deviation (RSD) > 30% of QC samples were removed, and log10 logarithmization was performed to obtain the final data matrix (Kaleem et al., 2022). After data treatment, the collected data were analyzed using principal components analysis (PCA), variable influence in projection (VIP), and the Student's *t*-test (*P* < 0.05) in sequence.

### Comparative analysis of antimicrobial activity between metabolites and strain 5–4 extracts

Antimicrobial activity was determined using an agar-well diffusion method (Wonglom et al., 2019). The extracts were added to the sterilized PDA medium with a final concentration of 2.5 to 200 μg/mL. After solidification, a fungal disk of 5 mm diameter was inoculated on the PDA plate. All experiments were performed in triplicate. The antimicrobial activity was measured by the inhibition rate (Cao et al., 2022). The half-maximal effective concentration (EC_50_) was calculated from the toxicity regression equation. The mycelium characteristics of Foc TR4 treated with strain 5–4 extracts were observed under a microscope.

### Statistical analysis

Statistical analysis was conducted using the SPSS software. The data were expressed as the means ± SE and analyzed using one-way ANOVA. Significance was tested using Tukey's HSD test. Statistically significant differences were determined at a *p*-value of < 0.05.

## Results

### Potent and cidal action against Foc TR4 by strain 5–4

**Figure 1:**
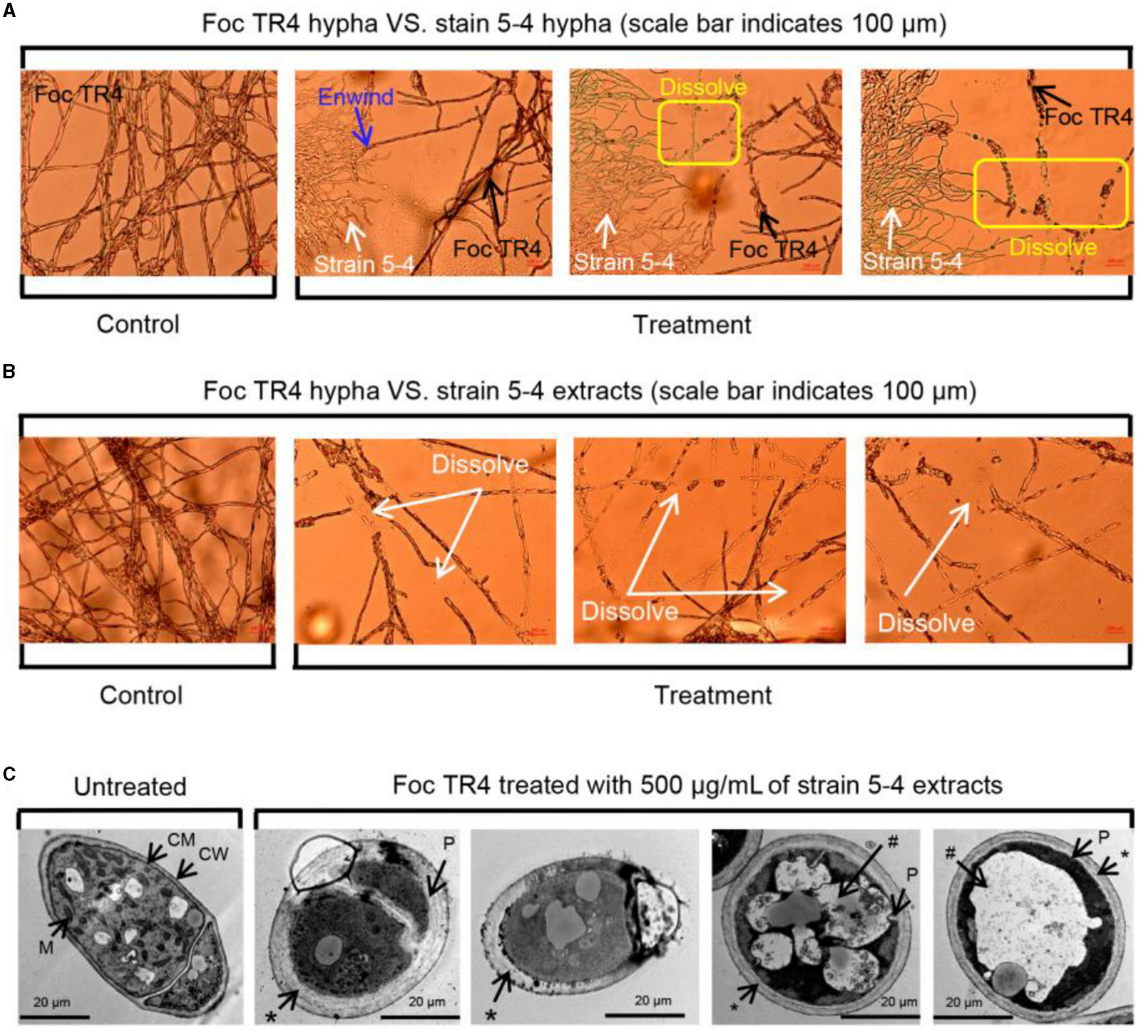
Potent and cidal action of strain 5–4 against Foc TR4 *in vitro*. **(A)** Antagonistic effects of strain 5–4 on the mycelial development of Foc TR4. **(B)** Extracts of strain 5–4 cause the disintegration of Foc TR4 mycelium. **(C)** Ultrastructure of Foc TR4 mycelial treated with extracts; mitochondria (M), cell membrane (CM), cell walls (CW), thickened cell walls (^*^), plasmolysis (P), and vacuolization (#).

The inhibitory ability of strain 5–4 on the mycelial growth of Foc TR4 was visualized using light microscopy and TEM. A confrontation culture assay showed that strain 5–4 exhibited intertwining growth with the Foc TR4 mycelium. Additionally, clear signs of lysis were evident at the contact site between their respective mycelia (Figure 1A). We speculated that the lysis of Foc TR4 mycelium was caused by the metabolites of strain 5–4. Therefore, we studied the effect of strain 5–4 extract at a concentration of 500 μg/mL on the mycelia of Foc TR4. Upon examination of the effect of extracts on the mycelia morphology of Foc TR4 under the light microscope, it was observed that the treated Foc TR4 mycelium experienced significant rupture and disintegration. In contrast, the control mycelia maintained a normal morphology (Figure 1B). Therefore, the inhibitory effect of strain 5–4 against Foc TR4 was mainly caused by its secondary metabolites. The effects of extracts on Foc TR4 cells were further studied using TEM (Figure 1C).

In untreated mycelia, normal intact fungal cell components such as cell walls (CW), cell membranes (CM), mitochondria (M), and vesicles (V) were observed to be normal and uniformly distributed. However, after treatment with extracts, notable changes were observed in the Foc TR4 mycelia. The cell wall and membrane of Foc TR4 mycelia exhibited irregular thickening, forming a multilayered cell wall, with the outer wall showing fibrous digestion. The cytoplasm was reduced, concentrated, and clearly vacuolated. The organelles within the cells were difficult to discern, with most of them damaged and undergoing hydrolysis. These results suggest that the antagonistic metabolites penetrated the cytoplasm of Foc TR4 cells and destroyed their organelles.

### Effect of strain 5–4 extracts on the cell membrane of Foc TR4

To investigate the interfering effect of strain 5–4 extracts on the Foc TR4 cell membrane function, we determined the effects of strain 5–4 extracts on the content of SP, ERS, NOX, and MDA of Foc TR4.

**Figure 2:**
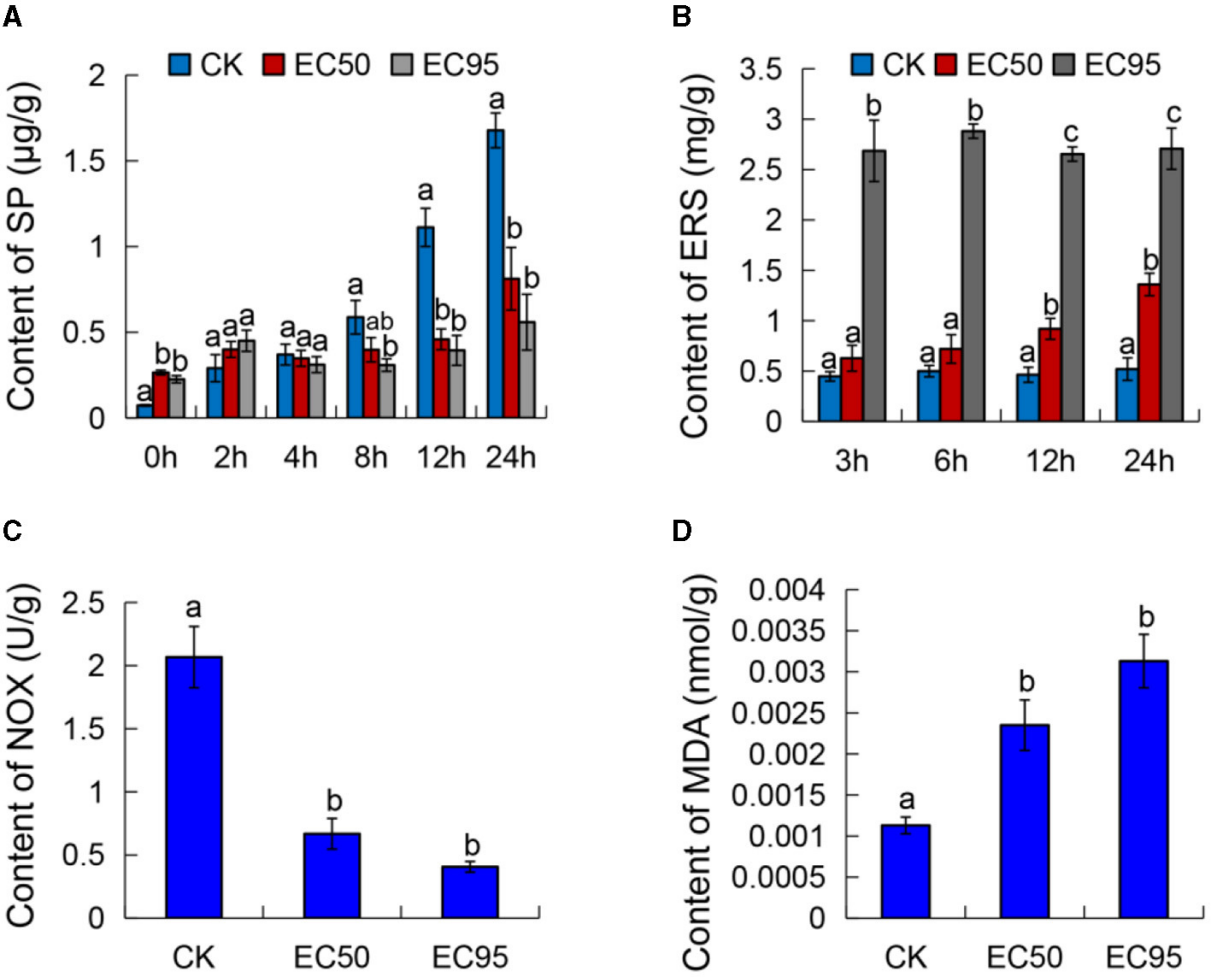
Effect of extracts on the cell membrane of Foc TR4. **(A)** The content of soluble protein (SP), **(B)** extracellular reducing sugar (ERS), **(C)** NADH oxidase activity (NOX), and **(D)** malondialdehyde (MDA) of Foc TR4. Different letters were used to indicate significant differences (*p* < 0.05).

The SP content of Foc TR4 was reduced significantly after exposure to different concentrations of extracts compared with the control (Figure 2A). The SP content in both the controls showed a rapidly rising trend with the increase in cultured times; the maximum value was 1.68 μg/g at 24 h. The SP content of EC_50_ and EC_95_ treatments slowly increased with culture time. After 24 h of exposure to the extracts, the SP contents measured 0.81 and 0.56 μg/g at the EC_50_ and EC_95_ concentrations, respectively. These values were considerably lower compared to the control group.

The ERS content of Foc TR4 increased considerably after exposure to different concentrations of extracts, while the ERS content remained stable in the untreated cells (Figure 2B). The content of ERS at the EC_50_ and EC_95_ concentrations were all higher than the control, and this change became more apparent with an increase in exposure times. At 24 h of exposure to the extracts, the ERS content of Foc TR4 treated with EC_50_ and EC_95_ concentrations were 1.36 and 2.71 mg/g, which were significantly higher than the control (0.52 mg/g). These results suggested that extracts may lead to cell membrane injury in Foc TR4 and cause ERS leakage.

The activities of NADH oxidase decreased with the increased extract concentration and showed an apparent gradient (Figure 2C). NADH oxidase activities of Foc TR4 were 0.668 and 0.41 U/g when treated with the EC_50_ and EC_95_ concentration extracts, respectively. The activities were significantly lower compared to the control group (2.067 U/g). The results showed that the NADH oxidase activity of Foc TR4 decreased in a concentration-dependent manner with an increase in the concentration of extracts.

To further investigate the damage of extracts to the Foc TR4 cell membrane, we detected the MDA content, which is often used as an index of the oxidative injury of the cell membrane (Liu et al., 2021). Figure 2D showed that the MDA content of the control was 0.00113 nmol/g. When exposed to the extracts of EC_50_ and EC_95_ concentrations, the MDA contents were significantly higher compared to the control, measuring 0.00235 and 0.00313 nmol/g, respectively. These findings demonstrated a dose-dependent increase in the MDA content of Foc TR4 as the concentration increased. These results confirmed that the cell membrane of Foc TR4 caused irreversible damage after treatment with the extracts, suggesting a direct interaction between the metabolites of strain 5–4 and the components of the membrane.

### Effect of extracts on the mitochondrial membrane potential of Foc TR4

An early indication of cell apoptosis is the reduction of mitochondrial membrane potential, which can be detected by observing the fluorescence intensity produced by cell dyes (Wu et al., 2022). The fluorescence intensity of rhodamine showed a significant reduction after the EC_95_ treatment. Compared with the control, the mitochondrial membrane potential decreased (Figure S1). The mitochondrial membrane potential showed a positive correlation with the concentrations of the extract.

### Effect of extracts on mitochondrial respiratory chain complex enzyme activity of Foc TR4

**Figure 3:**
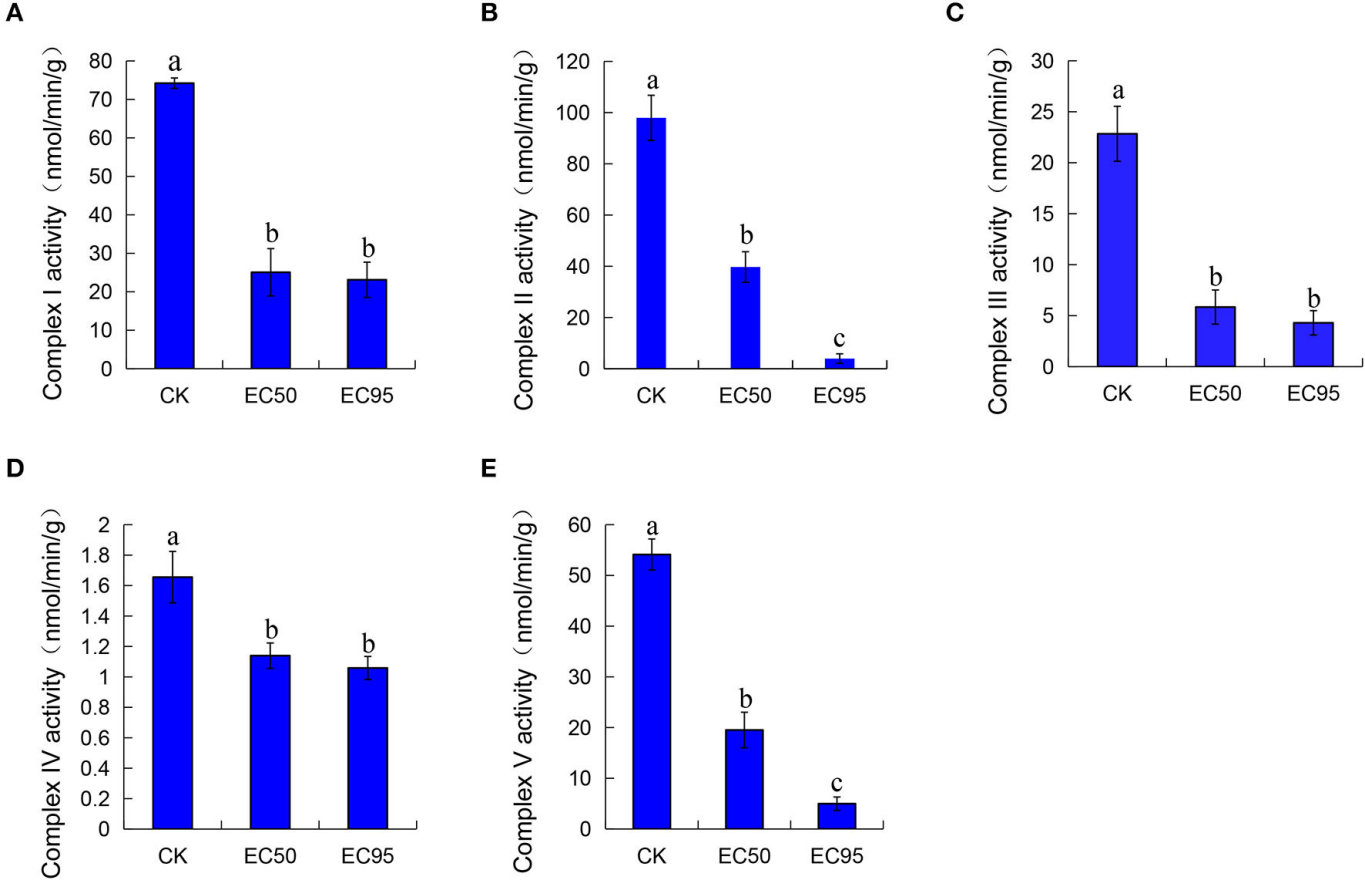
Effect of extracts on the mitochondrial respiratory chain activity complex I **(A)**, complex II **(B)**, complex III **(C)**, complex IV **(D)**, and complex V **(E)** of Foc TR4. Compared with the control groups, the groups of EC_50_ and EC_95_ showed dramatic reductions in the activity of complex I-V by approximately 30–90%. Different letters were used to indicate significant differences (*p* < 0.05).

The mitochondrial respiratory chain can provide 95% of the energy for cellular life activities and is mainly carried out by mitochondrial respiratory chain enzymes. The respiratory chain consists of five complexes located on the inner mitochondrial membrane (Anamika et al., 2021; Tang et al., 2021). The mitochondrial respiratory chain complex I-V activity of Foc TR4 after treatment with extracts for 30 min was determined. As shown in Figure 3, the activities of complex I-V decreased after extracts were treated compared with the control groups. At the concentration of EC_50_ treatment, the activity of complex I-V was 25.06, 39.70, 5.85, 1.14, and 19.52 nmol/min/g, which were significantly decreased compared with the control groups (74.22, 97.96, 22.84, 1.66, and 54.12 nmol/min/g). Treated with a high concentration of EC_95_ extracts, the activities of complex I-V were 23.12, 3.94, 4.30, 1.06, and 4.99 nmol/min/g, respectively. These results showed dramatic reductions in the activity of complex I-V by approximately 30–90% compared with the control groups.

### Variation tendency of strain 5–4 antimicrobial extracts during fermentation culture determined by HPLC-MS

To detect any antimicrobial activity of strain 5–4 extracts, a filter paper diffusion method was employed at different time points: 2, 4, 6, 7, and 8 days post-inoculated cultured (dpi). The antimicrobial activity of the extracts was 16.5, 20.64, 37.37, 41.26, and 33.79%, respectively (Supplementary Figure S2A). With the progression of fermentation cultures, the antimicrobial activity increased, and the antimicrobial activity of 7 dpi was the highest, which was significantly higher than that of 2 dpi. To investigate the metabolome changes of strain 5–4 extracts during fermentation culture, the metabolites of extracts obtained at 2 dpi, 4 dpi, and 7 dpi were analyzed using HPLC-MS.

Principal component analysis (PCA) was used to analyze the discrete intra- and inter-group differences in metabolites (Gao et al., 2022). In the PCA score plots, the principal components in ESI+ (Supplementary Figure S2B) and ESI- (Supplementary Figure S2C) modes accounted for 62.60% and 60.30% of the variation, respectively. The samples for the intra-group comparison were gathered, which showed the reliability and repeatability of the experiment.

**Figure 4:**
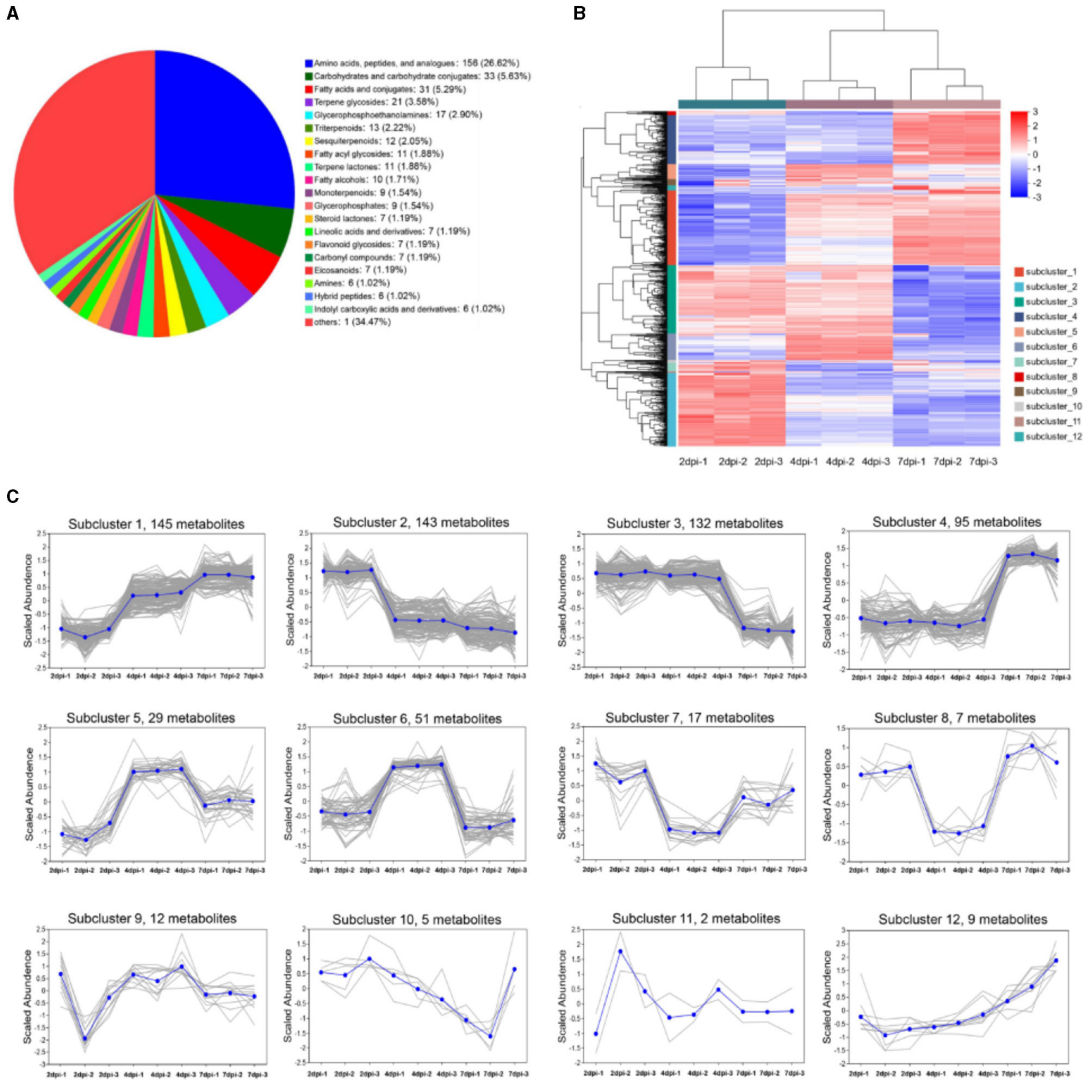
The metabolites of strain 5–4 were identified in three fermentation periods [2, 4, and 7 days post-inoculated cultured (dpi)]. Three biological replications were performed to analyze the metabolite profiling. **(A)** Pie chart of strain 5–4 metabolome data. A total of 647 metabolites were found in the subclass; **(B)** Hierarchical cluster analysis of metabolites in three fermentation periods. Divided into 12 subclusters; **(C)** K-MEAN analysis of metabolites. The subcluster 1 and 4 of metabolites were increased with fermentation periods.

In total, 647 metabolites in strain 5–4 extracts were identified at three fermentation periods, including “amino acids, peptides, and analogs” (156 metabolites, 26.62%); “carbohydrate metabolomics” (33 metabolites, 5.63%); “fatty acids and conjugates” (31 metabolites, 5.29%); “terpene glycosides” (21 metabolites, 3.58%); “glycerophosphoethanolamines” (17 metabolites, 2.90%); “triterpenoids” (13 metabolites, 2.22%); “sesquiterpenoids” (12 metabolites, 2.05%); “fatty acyl glycosides” (11 metabolites, 1.88%), and “terpene lactones” (11 metabolites, 1.88%) (Figure 4A). The results showed that the primary metabolites in strain 5–4 extracts were rich and diverse. Abundant terpenoids and amino acid compounds were observed in the secondary metabolites, with a few compounds being “steroidal glycosides” and “glycosphingolipids.” In addition, several hormones and hormone analogs were identified, such as flavonoid glycosides and indoles (Table S1). The hierarchical cluster analysis showed that the metabolites were significantly separated at different fermentation periods (Figure 4B). These metabolites were divided into 12 subclusters using K-MEAN analysis (Figure 4C). Subcluster 1 contained the most metabolites (145), and subcluster 11 had only two metabolites. The relative contents of metabolites in the subcluster 1 and 4 increased with an extension of fermentation periods. Therefore, the key antimicrobial metabolites existed in subclusters 1 and 4.

**Figure 5:**
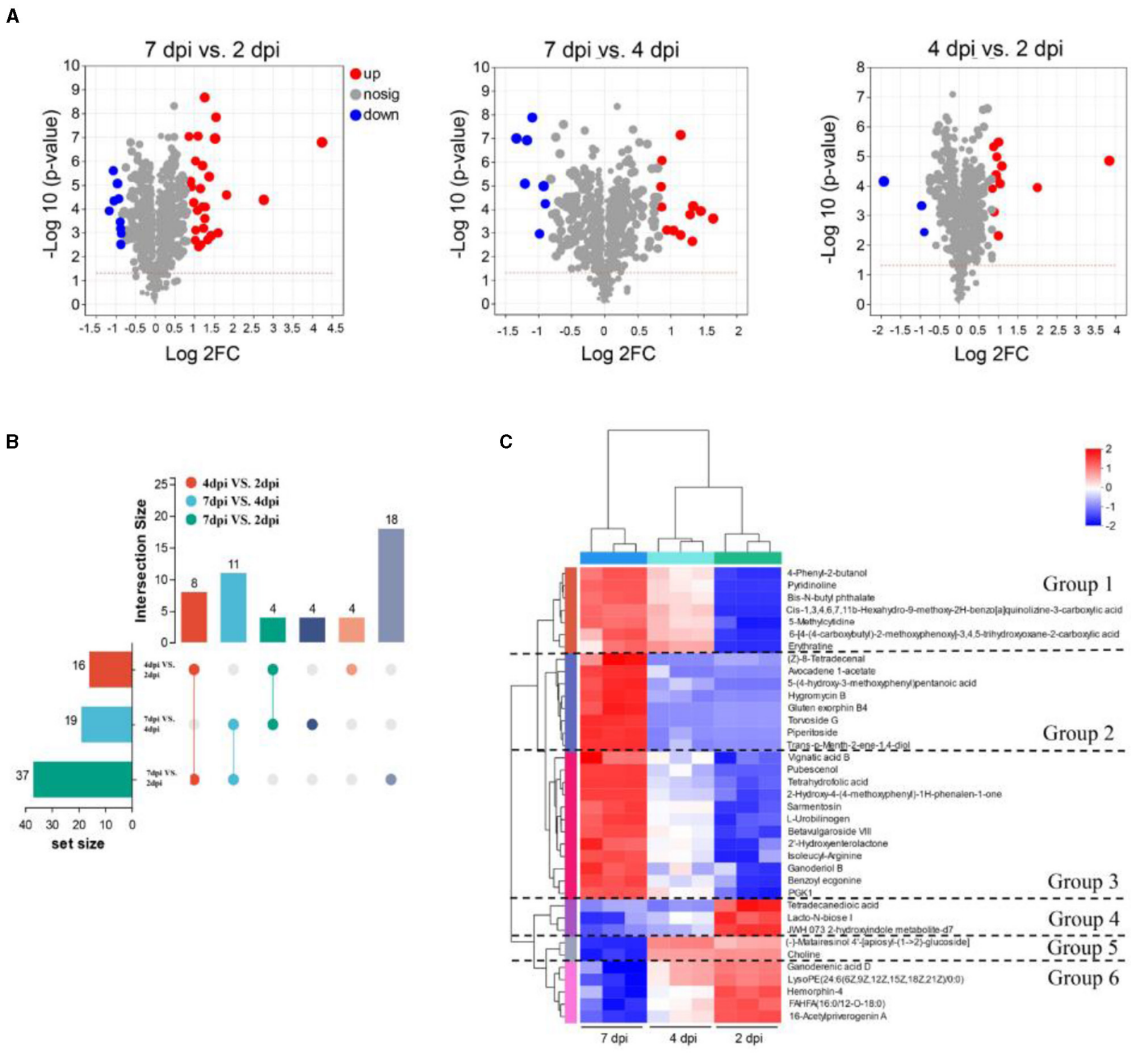
Analysis of the differentially accumulated metabolites (DAMs) at three fermentation periods. **(A)** Volcano plot of DAMs. Each dot represents a metabolite, blue dots are downregulated DEMs, red dots are upregulated DEMs, and gray dots are no significantly different metabolites. **(B)** The number of DAMs at three fermentation periods. The left column shows the number of DAMs in different pairwise comparisons, and the right column represents overlapping and specific DAMs in different comparisons. **(C)** The heatmap analysis of 37 DAMs in the three fermentation periods. The metabolites are generated in six groups.

Metabolites with VIP > 1 and FC = > 1.8 were defined as differentially accumulated metabolites (DAMs). As a result, the 37 DAMs (27 upregulated, 10 downregulated) were identified in groups of 7 dpi vs. 2 dpi, the 19 DAMs (12 upregulated, 7 downregulated) in groups of 7 dpi vs. 4 dpi, and the 16 DAMs (13 upregulated, 3 downregulated) in groups of 4 dpi vs. 2 dpi (Figure 5A). The upregulated metabolites of strain extracts at 7 dpi were mainly triterpenoids, amino acids, carbohydrates, and their derivatives (Supplementary Table S2). UpSet Venn diagram analysis revealed that the 18 specific DAMs were identified in groups of 7 dpi vs. 2 dpi. Ganoderenic acid D, pubescenol, tetrahydrofolic acid, hemorphin-4, vignatic acid B, and L-urobilinogen belonged to the class. The groups of 7 dpi vs. 4 dpi and 7 dpi vs. 2 dpi shared 11 DAMs and contained hygromycin B and piperitoside (Figure 5B). A heat map cluster analysis was conducted on the DAMs, and six groups were generated. The DAMs of groups 2 and 3 may be the key antimicrobial metabolites, which are only significantly enriched at 7dpi (Figure 5C). To explore the physiological processes of these DAMs, KEGG annotation, and analysis were conducted. A total of 100 pathways were involved in the DAMs (Supplementary Table S3). The pathways of “biosynthesis of plant secondary metabolites” and “protein digestion and absorption” were associated with the most DAMs. The KEGG pathway enrichment analysis showed that “novobiocin biosynthesis,” “biosynthesis of vancomycin group antibiotics,” “zeatin biosynthesis,” “carbapenem biosynthesis,” “bacterial chemotaxis,” “biosynthesis of plant hormones,” “tropane, piperidine, and pyridine alkaloid biosynthesis,” and “Staurosporine biosynthesis” were significantly enriched (*p* < 0.05) (Supplementary Figure S3A). To identify unique DAMs at 7 dpi, VIP values, and *p*-values were used for screening. The predictive performance of these DAMs were evaluated using receiver operating characteristic curves (ROC). DAMs with an AUC of 1 were considered to have good predictive performance and were selected as potential biomarkers. Notable DAMs with an AUC of 1 included hygromycin B, gluten exorphin B4, tetrahydrofolic acid, torvoside G, and pipertoside (Supplementary Figure S3B). An abundance of hygromycin B at 7 dpi was significantly higher compared to that at 2 dpi (Supplementary Figure S3C). This suggests that hygromycin B may be a key metabolite responsible for the antimicrobial activity exhibited by strain 5–4.

### Comparative analysis of antimicrobial activity between hygromycin B and strain 5–4 extracts

**Figure 6:**
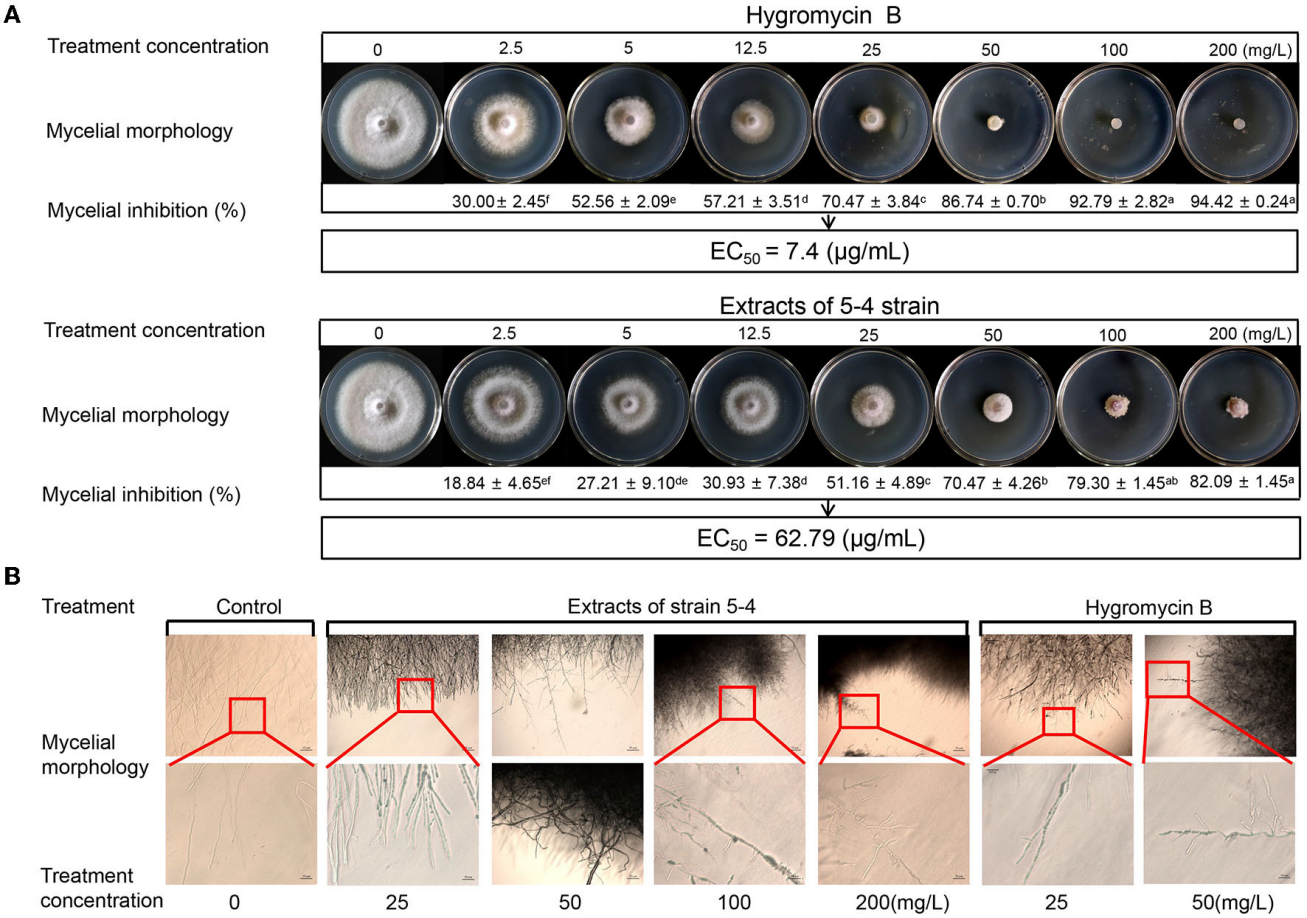
Antagonistic effects of hygromycin B and strain 5–4 extracts against Foc TR4 *in vitro*. **(A)** Antimicrobial activity of Foc TR4 treated with various concentrations of hygromycin B and strain 5–4 extracts (0, 2.5, 5, 12.5, 25, 50, 100, and 200 mg/L). Different letters were used to indicate significant differences (*p* < 0.05). **(B)** The hindered growth of Foc TR4 mycelium in the presence of hygromycin B and strain 5–4 extracts with microscopic observation.

The inhibitory effects of various concentrations of hygromycin B and extracts on the growth of Foc TR4 mycelium are depicted in Figure 6. In the control group, colony growth continued to increase during cultivation at 28°C. However, hygromycin B and extracts (2.5–200 μg/mL) demonstrated antifungal activity against Foc TR4, exhibiting a dose-dependent effect. Compared to the control group, the antifungal activity of extracts and hygromycin B at a concentration of 200 μg/mL was 82.09 ± 1.45% and 94.42 ± 0.24%, respectively (Figure 6A). Using the log-transformation analysis calculation, we found that the EC_50_ value of hygromycin B against Foc TR4 was 7.4 μg/mL. Moreover, when Foc TR4 mycelium was treated with hygromycin B and extracts from strain 5–4, noticeable coarseness and abnormal branching of the mycelium were observed (Figure 6B). These results indicated that both hygromycin B and strain 5–4 extracts had similar effects on the morphology of Foc TR4 mycelium.

## Discussion

Bananas are an important fruit for production and global trade (Dita et al., 2018). However, the prevalence, severity, and rapid spread of FWB have led to substantial economic losses for banana farmers and posed a serious threat to the development of the banana industry (Dusunceli, 2017). Compared with traditional chemical pesticides, biological control has advantages such as eco-friendly nature and low investment costs. Biocontrol has shown promising effectiveness in managing FWB (Bubici et al., 2019). The effects of different bioagents can vary depending on the specific microbes and the complex natural environment. Therefore, it remains crucial to adapt to local environments and develop safer and more effective biological agents. This approach holds significant guiding importance for practical production purposes. Endophytic actinomycetes have been reported as a major source of bioactive metabolites and are considered a promising strategy for the biocontrol of plant pathogens (Xu et al., 2020).

Strain 5–4 were isolated from the roots of *Piper austrosinense* and exhibited high antimicrobial activity against Foc TR4. In our present study, the antifungal mechanisms and metabolomic profile of strain 5–4 were analyzed. Consistent with the results of previous studies, the morphological changes in Foc TR4 mycelium were observed through microscopic analysis. Therefore, comparing the potency of the mycelium's growth can provide information on its mode of antimicrobial action. The results showed that strain 5–4 exhibited inhibitory effects on the mycelia development of Foc TR4, leading to malformation and dissolution of the mycelium. We believed that the antifungal mechanism of strain 5–4 extracts involved the disruption of the cell membrane and cell wall of Foc TR4, ultimately resulting in the death of the Foc TR4 cells. In our previous study, strain 5–4 destroyed the cell wall of Foc TR4 through the action of cell wall-degrading enzymes. Therefore, the secretion of β-1,3-glucanase and chitinase by strain 5–4 may be considered one of its antifungal mechanisms (Yun et al., 2022).

In addition to the cell wall, the cell membrane is the mechanical and osmotic barrier between the cell and the external environment and is a common action site of fungicides against pathogenic fungi (Kim and Lee, 2019; Xu et al., 2019; Zhang et al., 2021). Light microscope analysis showed that strain 5–4 caused the abnormally lysed Foc TR4 mycelium. Moreover, TEM analysis revealed that the cell membrane of Foc TR4 was damaged, and organelles were degraded. The changes in Foc TR4 mycelium may be related to an increase in cell permeabilization (Wang et al., 2019). Thus, the antifungal mechanism of strain 5–4 extracts against Foc TR4 might be dependent on cell membrane destruction, leading to the death of the cell (Wang et al., 2019; Chen et al., 2022a). The integrity of the cell membrane is essential for the growth of Foc TR4. Soluble proteins and reducing sugars are critical cellular components and fundamental to the function of fungi (Chen et al., 2020). The impact of strain 5–4 on the integrity of the Foc TR4 cell was evaluated by determining the contents of soluble proteins and reducing sugar. The results showed a significant decrease in the content of SP after exposure to different concentrations of extracts, suggesting leakage of endoplasmic reticulum (ER) stress in the Foc TR4 cells compared to the control groups. NADH oxidase activity is a vital antioxidant function that is the main defense barrier for protecting cellular metabolism (Li et al., 2022). The content of MDA is commonly used as an indicator of oxidative damage to the cell membrane (Liu et al., 2021). The results indicated a dose-dependent decrease in NADH oxidase activities of Foc TR4, along with a dose-dependent increase in MDA activities, following treatment with extracts of strain 5–4. These results confirmed that the cell membrane of Foc TR4 was irreversibly damaged after being exposed to the extracts of strain 5–4. This suggests that the metabolites produced by strain 5–4 may directly interact with the components of the cell membrane.

Mitochondria are one of the earliest and most sensitive organelles for sensing biological damage (Zhang et al., 2021). The decreased fluorescence intensity of rhodamine-123 indicates that the extract of strain 5–4 caused a reduction in the mitochondrial membrane potential of Foc TR4. The activity of the mitochondrial respiratory chain complex enzymes plays an essential role in organisms, with complex II being one of the most important targets in the field of pesticide research, especially for the discovery of novel fungicides (Zhang and Chen, 2017; Zhao et al., 2020). The activity of complex I-V was observed to decrease after treatment with the extracts, displaying a negative correlation with the dose and a significant difference. Inhibition of electron transport in the mitochondrial respiratory chain disrupts the normal flow of electrons, leading to the accumulation of free radicals. The extent of free radical formation depends on the level of panthenol (Chen et al., 2022b). The accumulation of free radicals caused by the altered activity of complex III may be responsible for the extract-induced production of ROS in Foc TR4.

When the cell membrane of fungi is destroyed, it causes the efflux of cellular contents such as cytoplasm and proteins. This disruption also leads to the disorder of transmembrane potential, causing irreversible damage to the cell membrane and inhibiting the growth of fungal mycelium (Lin et al., 2018; Zhang et al., 2021). By targeting multiple sites and exerting various kinds of pressure, the stress response of the fungus becomes overwhelmed and unable to sustain growth. In the case of strain 5–4, it appears that the cell membrane of Foc TR4 is the main target, indicating its significant impact on fungal growth and development.

Metabolic profiles of strain 5–4 were performed using LC-MS analysis at different time periods, revealing changes in metabolite composition over the course of fermentation. The LC-MS analysis identified a total of 876 DAMs that exhibited an increase in the group of 7 dpi, including several unknown metabolites. These findings indicate the ability of strain 5–4 to produce a diverse range of metabolites during the fermentation process. Among the secondary metabolites, an abundance of terpene and amino acid compounds was observed, along with a few steroidal glycosides and glycosphingolipids. Notably, 27 upregulated DAMs were identified in the group of 7 dpi compared to 2 dpi. Furthermore, a high abundance of DAMs was involved in 100 pathways, with significant clustering in the biosynthesis of plant secondary metabolites and the protein digestion and absorption pathway. The KEGG pathway enrichment analysis showed significant enrichment of pathways such as novobiocin biosynthesis, biosynthesis of vancomycin group antibiotics, and zeatin biosynthesis. Among the unique DAMs identified in the 7dpi group based on VIP and p-value values, hygromycin B, gluten exorphin B4, tetrahydrofolic acid, torvoside G, and pipertoside were prominent. Comparative analysis of the antimicrobial activity between hygromycin B and strain 5–4 extracts showed similar inhibitory effects on the mycelium of Foc TR4. Hygromycin B belongs to the aminoglycoside antibiotic family and is known to inhibit protein synthesis in both prokaryotes and eukaryotes (Li et al., 2020). Therefore, we speculate that hygromycin B could target the cell membrane for Foc TR4, thereby inhibiting its growth. Hence, hygromycin B may be considered the key metabolite responsible for the antimicrobial activity of strain 5–4.

Based on the results of this study, it was determined that the antifungal mechanism of strain 5–4 inhibited the mycelium of Foc TR4 mycelium growth through the damage inflicted on the cell membrane and the suppression of mitochondrial respiratory chain complex enzyme activity. In future studies, we should explore the specific mechanisms of action of hygromycin B and determine the presence of other potentially significant antifungal metabolites. Such research will pave the way for the development of highly potent fungicides with enhanced efficacy for agricultural applications.

## Conclusion

*Streptomyces hygroscopicus* subsp. *hygroscopicus* 5–4 exhibited strong antifungal activity against Foc TR4. The most possible antimicrobial mechanism of strain 5–4 was explored using metabolomics, light microscopy imaging, and TEM. The morphological alterations in Foc TR4 mycelium cleared, indicating rupture and damage. Moreover, the organelles of Foc TR4 exhibited significant damage and hydrolysis. Strain 5–4 effectively inhibited the growth of Foc TR4 by targeting the cell membrane, resulting in the release of cellular contents and impairment of mitochondrial function. These multifaceted pressures on different cellular sites overwhelmed the fungal stress response system, leading to the inability to sustain growth. These results indicate that the direct antagonistic activity of strain 5–4 against Foc TR4 might be due to its damage to the integrity of the Foc TR4 cell membrane. Strain 5–4 produced an abundance of metabolites during the fermentation period and exhibited strong antifungal activity against Foc TR4 at 7 dpi. VIP and *p*-value values, the unique DAMs identified at 7 dpi included hygromycin B, gluten exorphin B4, tetrahydrofolic acid, torvoside G, and pipertoside. Hygromycin B and strain 5–4 extracts exhibited similar inhibitory effects on the mycelium morphology of Foc TR4. Hence, hygromycin B may be the key antimicrobial metabolite of strain 5–4.

## Data availability statement

The original contributions presented in the study are included in the article/Supplementary material, further inquiries can be directed to the corresponding authors.

## Author contributions

TY and TJ conceived the research. JX supervised the research. KL, YZ, and XZ performed some experiments. TY and DZ analyzed the data. TY and WW prepared the manuscript. All authors contributed to the article and approved the submitted version.
