# Supplementary material for: Antimicrobial mechanisms and secondary metabolite profiles of Streptomyces hygroscopicus subsp. hygroscopicus 5–4 against banana fusarium wilt disease using metabolomics
Source: Front Microbiol. 2023 Jun 9;14:1159534. doi: 10.3389/fmicb.2023.1159534 (PMC10289025; doi:10.3389/fmicb.2023.1159534)
Supplement: Supplementary file 1 [file Data_Sheet_1.docx]

Supplementary Material

## Supplementary Figure.


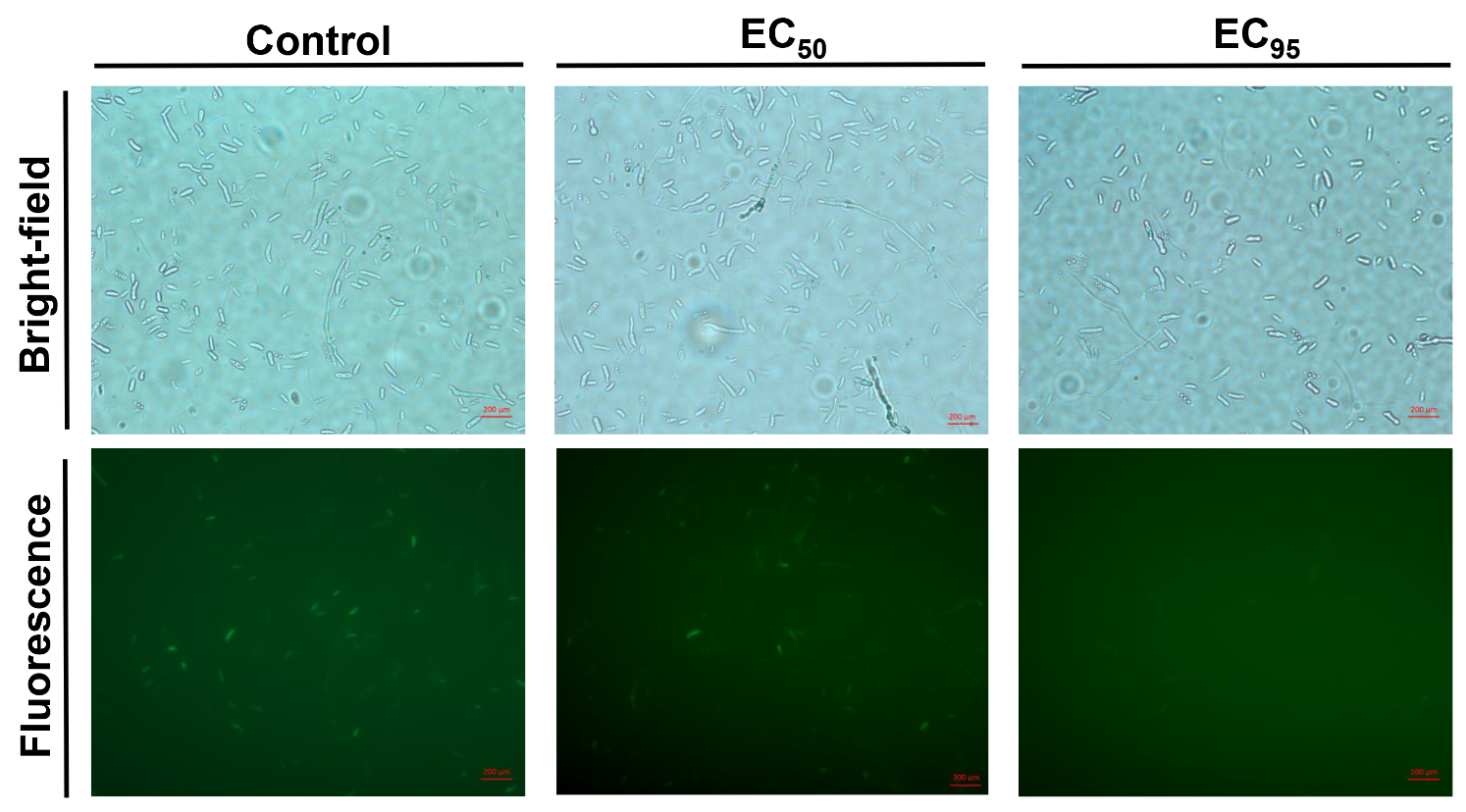


**Figure S1 | Effect of extracts on mitochondrial membrane potential of Foc TR4. EC50, the concentration was 24.73 μg/mL; EC95, the concentration was 869.94 μg/mL; control, sterile water treatment. The scales were 200μm.**


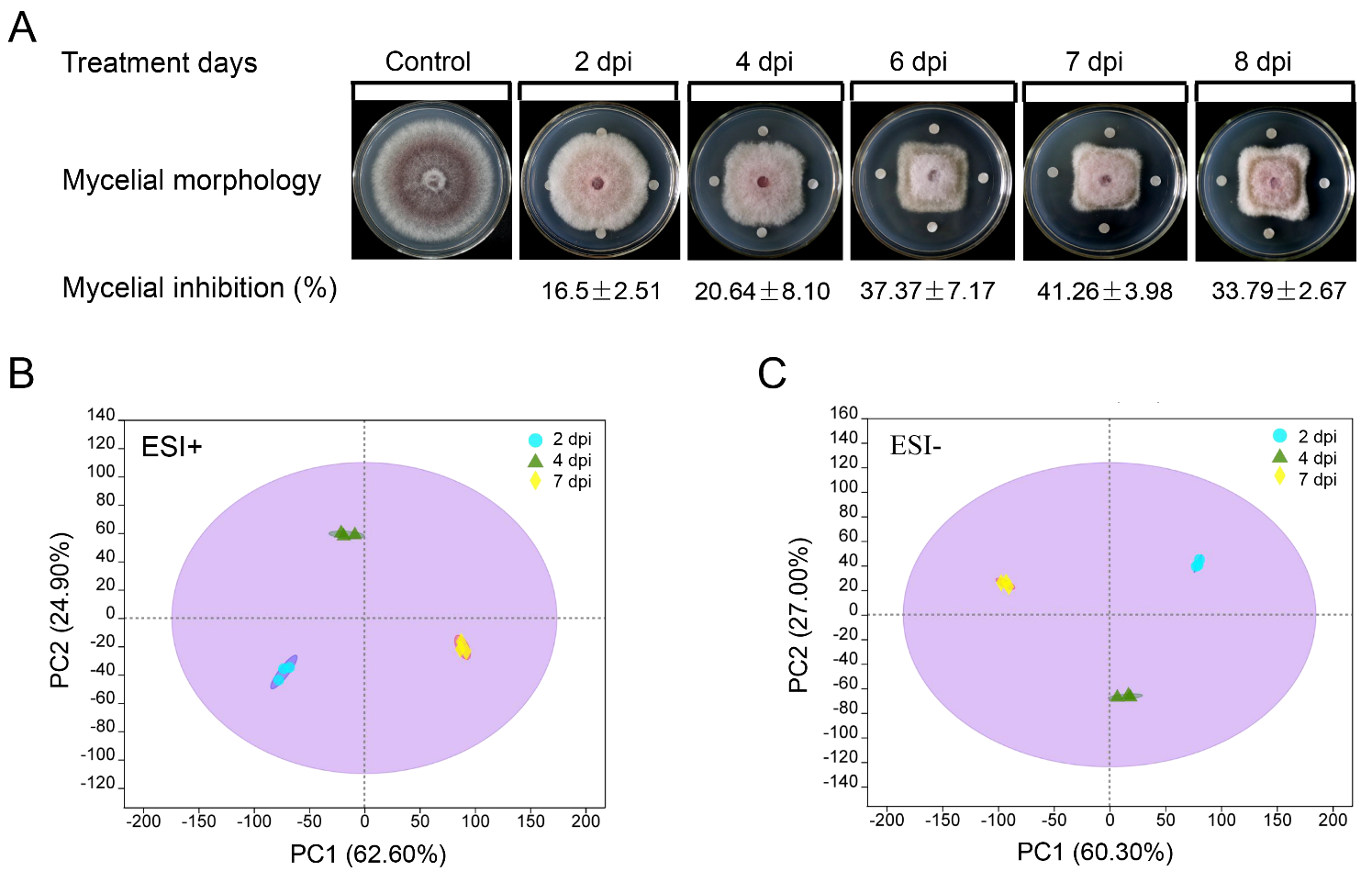


**Figure S2 | Variation tendency of strain 5-4 antimicrobial extracts during fermentation culture (A) and PCA score of strain 5-4 extracts during three fermentation periods in positive (B) and negative (C) ion modes.**


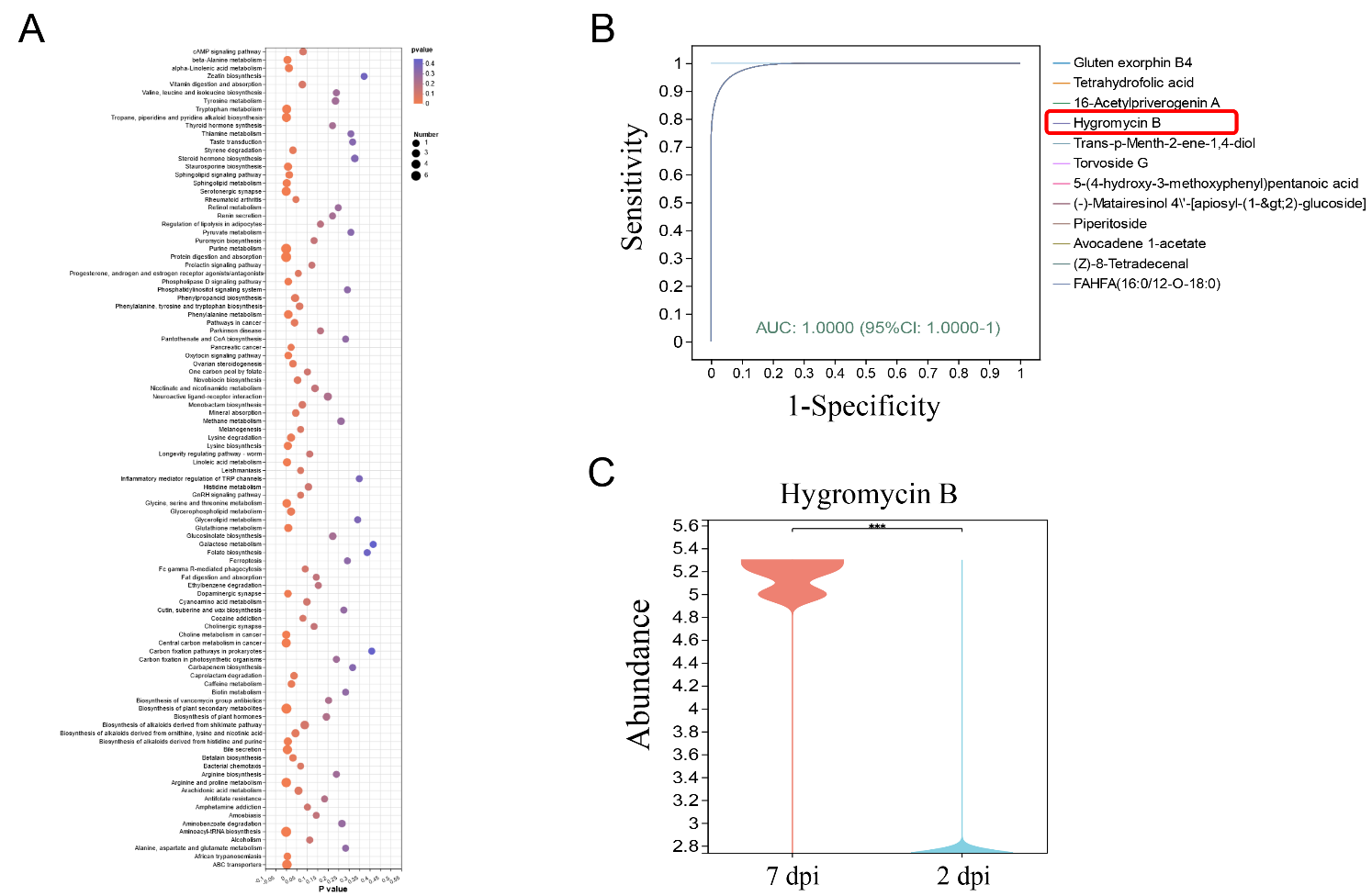


**Figure S3 | Analysis of strain 5-4 on differentially accumulated metabolites (DAMs). (A) Pathway enrichment analysis of significant differences metabolites of strain 5-4. (B) Receiver operating characteristic curves (ROC) of DAMs between three fermentation periods with AUC=1. (C) Compare the abundance with hygromycin B in 7 dpi and 2dpi.**
